# Pain catastrophizing is associated with reduced neural response to monetary reward

**DOI:** 10.3389/fpain.2023.1129353

**Published:** 2023-09-07

**Authors:** Megan E. Cooke, Robert R. Edwards, Grace L. Wheeler, William A. Schmitt, Lindsay V. Nielsen, Joanna M. Streck, Randi M. Schuster, Kevin Potter, A. Eden Evins, Jodi M. Gilman

**Affiliations:** ^1^ Center for Addiction Medicine, Department of Psychiatry, Massachusetts General Hospital, Boston, MA, United States; ^2^ Athinoula A. Martinos Center in Biomedical Imaging, Department of Radiology, Massachusetts General Hospital, Charlestown, MA, United States; ^3^ Harvard Medical School, Boston, MA, United States; ^4^ Department of Anesthesiology, Perioperative, and Pain Medicine, Brigham & Women’s Hospital, Chestnut Hill, MA, United States; ^5^ Tobacco Research and Treatment Center, Division of General Internal Medicine, Department of Medicine, Massachusetts General Hospital, Boston, MA, United States

**Keywords:** monetary incentive delay, depression, pain, fMRI, caudate, putamen

## Abstract

**Introduction:**

Pain catastrophizing, a measure of an individual's negative emotional and cognitive appraisals of pain, has been included as a key treatment target in many psychological interventions for pain. However, the neural correlates of pain catastrophizing have been understudied. Prior neuroimaging evidence suggests that adults with pain show altered reward processing throughout the mesocorticolimbic reward circuitry.

**Methods:**

In this study, we tested the association between Pain Catastrophizing Scale (PCS) scores and neural activation to the Monetary Incentive Delay (MID) reward neuroimaging task in 94 adults reporting a range of pain, insomnia, and mood symptoms.

**Results:**

Results indicated that PCS score but not pain intensity was significantly associated with blunted activation in the caudate and putamen in response to feedback of successful vs. unsuccessful trials on the MID task. Mediation analyses indicated that PCS score fully mediated the relationship between depression symptoms and reward activation.

**Discussion:**

These findings provide evidence that pain catastrophizing is independently associated with altered striatal function apart from depression symptoms and pain intensity. Thus, in individuals experiencing pain and/or co- morbid conditions, reward dysfunction is directly related to pain catastrophizing.

## 1. Introduction

Millions of Americans suffer from pain, which is a defining characteristic of many medical and psychiatric conditions, and is associated with substantial morbidity, mortality and disability (1–3). Research suggests that fear appraisal (i.e., predictions or interpretations of sensory input as threatening) plays an important role in the experience of pain (4–9). For example, studies have demonstrated that appraisal of innocuous sensory input as painful leads to experiencing pain, which in turn leads to further appraisal of threat and subsequent further increased pain, resulting in a self-reinforcing feedback loop (10–12). The fear-avoidance model (13) suggests that individuals who experience acute pain might develop fear and avoidance behaviors if they associate certain activities or movements with pain. This fear can lead to a cycle of increased pain, disability, and emotional distress.

One important type of fear appraisal is pain catastrophizing, which is broadly conceptualized as an exaggerated negative cognitive or affective response during an actual or anticipated pain experience (e.g., magnification of negative outcome expectancies) (14–16). Pain catastrophizing is associated with a host of adverse clinical outcomes independent of pain intensity including amplified pain experiences (17), greater negative functional impact of pain (18), and poor treatment response in patients with chronic pain (19–21). Importantly, pain catastrophizing has been listed as a risk factor for the development of chronic pain (22). As a result, pain catastrophizing has been included as a key treatment target in many cognitive-behavioral, acceptance- and mindfulness-based psychological treatments for pain (4, 23–25).

The neurobiological mechanisms underlying pain catastrophizing are not fully understood, especially regarding reward processing. Existing neuroimaging evidence suggests that adults with chronic pain, particularly those with co-morbid depression, show altered reward processing throughout the mesocorticolimbic reward circuitry (26–34), however this relationship has not been examined with pain catastrophizing. The relationship between pain catastrophizing and neural reward processes is critical for investigation because dysfunction in the mesocorticolimic system may indicate a mechanism through which pain catastrophizing exacerbates pain and poorer quality of life and thus could act as a neural target for treatment. Generally, pain catastrophizing is associated with heightened pain perception, emotional distress, and negative affect. This negative emotional state could potentially influence the brain's reward system and its response to rewarding stimuli. People who engage in pain catastrophizing might have difficulties experiencing rewards or positive emotions due to their heightened focus on pain and negative outcomes. Additionally, there is ongoing debate about whether pain catastrophizing is a distinct construct from depression given that both measure negative affect constructs (14). Some evidence suggests that pain catastrophizing is a distinct but related construct to depression and that pain catastrophizing mediates the relationship between depression and pain (14, 35, 36). However, no studies have examined whether depression and pain catastrophizing are associated with unique neural processes which is critical to begin parsing out these constructs as distinct vs. overlapping constructs in their contributions to pain.

Our group recently reported results of a clinical trial among a heterogeneous group of participants with pain, insomnia, and anxiety/depression symptoms who were randomized to medical cannabis or a waitlist control condition (37). This trial included a comprehensive functional magnetic resonance imaging (fMRI) battery at baseline, in addition to self-reported pain measures. Here, we report a secondary analysis of baseline fMRI scans, in which we investigate neural mechanisms associated with pain catastrophizing. We aimed to (1) investigate the neural correlates associated with pain catastrophizing among adults using baseline neuroimaging data from the parent clinical trial and (2) to examine the extent to which pain catastrophizing and depression display similar or different patterns of brain activation. We hypothesized that higher pain catastrophizing would be associated with decreased activation to monetary reward in the striatum, beyond the influence of more general affective factors such as comorbid depression symptoms.

## 2. Methods

### 2.1. Participants

This present study represents a secondary analysis of a larger randomized clinical trial of medical cannabis, (*NCT03224468*) and non-cannabis using community members with self-reported symptoms of pain, insomnia, depression, or anxiety. Participants were recruited primarily through community advertising. Exclusionary criteria included current heavy cannabis use (daily or almost-daily in the past 3 months), any magnetic resonance imaging (MRI) contraindications (e.g., pregnancy, claustrophobia), history of serious medical disorders (e.g., diabetes, cardiovascular disease, HIV, hepatitis C, migraines, head injury), as well as any current substance use disorder. All participants were enrolled between July 17, 2017 and February 11, 2020 and provided written informed consent. Screening procedures took place at Massachusetts General Hospital Center for Addiction Medicine, and scans were conducted at the Athinoula A. Martinos Center for Biomedical Imaging. This study was approved by the MassGeneral Brigham Human Research Committee.

### 2.2. Procedure

#### 2.2.1. Trial design

Eligible participants attended a baseline visit where they completed a battery of medical and psychiatric questionnaires. Participants also completed a baseline MRI scan at a subsequent session. During the baseline session prior to the MRI scan, participants completed a medical history, psychiatric interview [Mini-International Neuropsychiatric Interview; MINI (38)], and questionnaires assessing pain intensity, pain catastrophizing, and mood symptoms.

#### 2.2.2. Measures

##### 2.2.2.1. Brief pain inventory

The Pain Severity subscale of the Brief Pain Inventory Short Form (39) (BPI-PS) was used to assess pain intensity on a 0–10 point scale (0 = no pain, 10 = worst pain imaginable).

##### 2.2.2.2. Pain catastrophizing

The Pain Catastrophizing Scale (PCS) (40) consisted of 13 questions addressing feelings and thoughts related to the experience of pain (e.g., “There's nothing I can do to reduce the intensity of the pain.”). Each question was answered on a 5-point Likert scale with 0 being “not at all” and 4 being “all the time”, giving a total score ranging from 0 to 52. Higher scores indicated more catastrophizing.

##### 2.2.2.3. Depression

The Hospital Anxiety and Depression Scale (HADS) (41) assessed depressive (HADS-Dep) symptoms through 7 questions. Each question was answered on a 4-point Likert scale from 0 to 3, giving a total score ranging from 0 to 21. A score of 11 or higher indicated a probable mood disorder (depression) while a score of 8–10 indicated a borderline case.

#### 2.2.3. Neuroimaging

Participants were scanned using a 3 T Siemens Trio scanner with a 32-channel head coil. Structural and functional scans were acquired using parameters outlined by the Human Connectome Project (HCP) (42). A T1-weighted structural scan was acquired using the following parameters: TA = 7:38 min, voxel size = 0.7 mm^3^ × 0.7 mm^3^ × 0.7 mm^3^, GRAPPA acceleration factor 2, A-P phase encoding, 256 slices, 224 mm FoV read, slice thickness = 0.70 mm, TR = 2,400 ms, TE = 2.02 ms, TI = 1,000 ms, echo spacing 7.6 ms, bandwidth = 270 Hz/Px. Functional MRI data was acquired using the following parameters: TA = 8:47 min, voxel size = 2.0 mm^3^ × 2.0 mm^3^ × 2.0 mm^3^, slices = 69, phase encoding P-A, FoV read 220 mm, slice thickness 2.00 mm, TR = 1,530 ms, TE = 30.0 ms, Multi-slice mode = interleaved, slice acceleration factor PE = 2.

#### 2.2.4. Behavioral reward task

During the MRI scan, participants completed the monetary incentive delay (MID) task to measure reward anticipation and reward delivery of monetary rewards (43–45). There were three trial types: win money (small reward: $0.20 or large reward: $5.00), lose money (small punishment: $0.20 or large punishment: $5.00), or no incentive ($0). Participants saw a cue (pink circle, yellow square, or blue triangle) at the beginning of trials that indicated the valence (win/reward, loss/punishment, or no incentive) and the amount of money at stake ($0, $0.20, or $5.00). This cue presentation (2,000 ms) was followed by a jittered anticipatory delay (1,500–4,000 ms). A black target shape (matched shape as the previously presented cue) was then shown, and participants could gain money or avoid losing money by pressing a response button while the target shape was on the screen. The time the target was on the screen (150–500 ms) was dynamically manipulated to maintain a 60% success rate. After a short response window, feedback was provided (2,000 ms total). Participants received 20 reward trials, 20 loss trials, and 10 neutral trials. The adaptive algorithm resulted in 30 positive feedback trials (split between reward, loss, and neutral) and 20 negative feedback trials on average. Participants completed two runs of the task (approximately 5.5 min each). All subjects received practice on the task outside the MRI scanner and the mean reaction time (RT) from this practice was used to set the initial time the target was on the screen.

Performance was measured by calculating accuracy as well as reaction times. After the scan, participants filled out the post-MID questionnaire (PMQ) (43) which asks participants to rate the extent to which they felt vigorous, drowsy, energetic, excited, fearful, happy, quiet, restful, tense, tired, unhappy, or calm when they saw each trial type cue.

### 2.3. Analysis plan

#### 2.3.1. Demographic and behavioral outcomes

All effects in demographic, behavioral, and imaging outcomes were tested among all participants with baseline data (*n* = 94) and a useable MRI scan, regardless of recruitment source (clinical trial participants or participants from the community) to maximize statistical power. We investigated the association of PCS with (1) RT, (2) accuracy, and (3) responses on the PMQ by running a linear mixed effects model in R using the lme4 package. Due to the high correlations between emotions assessed in the PMQ, these emotions were averaged within trial type to create 3 broad mood states: positive (vigorous, energetic, excited), negative [drowsy, tired, fearful, unhappy, tense, happy (reversed coded)], and neutral (quiet, calm, restful). In all models, magnitude of MID trial type (small, large, neutral), valence of MID trial type (reward, punishment, neutral), and PCS score were included as fixed effects and participant was included as a random effect.

#### 2.3.2. fMRI data processing

Standard pre-processing procedures were performed in FSL (FMRIB's Software Library, www.fmrib.ox.ac.uk/fsl); each subject's functional and structural scans were first co-registered using FLIRT (FSL's linear registration tool) with functional registration using the BBR cost function with a fieldmap generated from two opposing polarity topup acquisitions, and in a next step registered to a high-resolution image in standard space. Motion correction was performed using MCFLIRT and brain extraction was performed for non-brain removal using BET (FSL's Brain Extraction Tool). Motion outliers were also removed using FSL's motion outlier command. Spatial smoothing was applied using a Gaussian kernel with a full-width at half maximum (FHWM) of 5 mm. A high-pass temporal filter was applied to remove lower frequency noise. Preprocessed images were analyzed using FEAT (FMRI Expert Analysis Tool) in FSL. A first-level within-subject analysis was performed using a general linear model (GLM). For each participant, contrast images of brain activity were generated for higher-level analyses. In a second-level between-subject analysis, regions of significant activation across the brain were assessed using permutation tests (*N* = 5,000) at a cluster threshold of 2.3 and a significance threshold of p < 0.05, with a reward mask obtained from NeuroSynth (reward_uniformity-test_z_FDR_0.01.nii; see Supplementary Figure S1) (46). Our main interest was the association between PCS and brain activation during the anticipation and feedback stage of the MID task. To differentiate the unique effects of pain catastrophizing and negative affect on reward processing, for any contrast with a cluster associated with PCS, we separately tested the association of PCS, BPI-PS, or HADS-Dep with voxels within the mask. In addition to the voxelwise analyses, we conducted ROI analyses using anatomical striatal masks created in WFUPickAtlas (http://fmri.wfubmc.edu/software/pickatlas) and extracted the mean contrast of parameter estimates (COPEs), or beta weights. The purpose of these analyses was to visualize and further explore effects seen in the voxelwise analyses. Pearson's correlations were calculated to investigate the relationship between change in brain activation within the ROI (win vs. loss trials), and clinical symptoms (PCS, BPI and HADS-Dep scores). ROI analyses were performed in R.

As a specificity analysis, we ran a set of mediation models comparing the extent to which PCS mediated the relationship between HADS-Dep and striatal activation or HADS-Dep mediated the relationship between PCS and striatal activation. Mediation analyses were performed in R using the mediation package (47).

## 3. Results

### 3.1. Demographics

Ninety-four participants who had usable baseline scans were included in these analyses. One participant was dropped due to poor scan quality and one was excluded due to a diagnosis of cannabis use disorder at baseline (exclusionary criteria for the parent study). The sample was primarily white and female, with 78% either full time employed or a student, and half the sample met diagnostic criteria for lifetime major depressive or anxiety disorder (See Table 1 for baseline participant characteristics).

**Table 1:** Sample demographics and descriptives.

Measure	Mean or *N* (SD or %)	Range
Age	36.38 (13.78)	
Sex—Female	64 (68.09%)	
Race		
Caucasian	76 (80.85%)	
African American	8 (8.51%)	
Asian	6 (6.38%)	
Multi-racial	2 (2.13%)	
Pacific Islander	1 (1.06%)	
Unknown	1 (1.06%)	
Right-handed	83 (88.30%)	
Full-time employed	56 (59.57%)	
Student	17 (18.08%)	
Education years	17.10 (2.88)	
Pain medications		
NSAIDs	28 (29.79%)	
Corticosteroids	8 (8.51%)	
Muscle relaxants	8 (8.51%)	
Opioids	6 (6.38%)	
Opiate antagonists	2 (2.13%)	
Triptan	2 (2.13%)	
Non-opioid/non-NSAID	18 (19.15%)	
Depression diagnoses		
Current MDD	8 (8.51%)	
Past MDD	40 (42.55%)	
Medical diagnoses/complaint*		
Arthritis	7 (7.45%)	
Migraines	25 (26.60%)	
Vertigo	11 (11.70%)	
GERD	13 (13.83%)	
Back pain	6 (6.38%)	
Knee pain	5 (5.32%)	
Shoulder pain	7 (7.45%)	
Anemia	6 (6.38%)	
BPI-severity	1.17 (1.92)	0–7.75
PCS	7.99 (9.64)	0–40
HADS-dep	3.83 (3.79)	0–14

MDD, major depressive disorder, PCS, pain catastrophizing scale, BPI, brief pain inventory, HADS-Dep, hospital anxiety and depression scale—depression.

* Medical conditions/complaint is based on self-report. Conditions endorsed by less than 5 subjects are not reported.

### 3.2. Correlations

At baseline, pain catastrophizing score (PCS) correlated with depression (*r* = 0.53, *p* = 4.08 × 10^−8^) but was not significantly associated with pain severity (BPI-PS, *r* = 0.198, *p* = 0.06).

### 3.3. Behavioral responses to the MID task

Reaction times (RTs) were significantly shorter for reward (*M* = 259, SD = 19.9) vs. loss (*M* = 261, SD = 20.7) or neutral trials (*M* = 266, SD = 23.7, *p* = 0.04) and were significantly shorter for large (±$5, *M* = 258, SD = 19.1) vs. small (±$0.20, *M* = 262, SD = 21.2) or no incentive trials (*M* = 266, SD = 23.7, *p* = 1.74 × 10^−7^). There was no significant association between PCS and RT (*p* = 0.31). Accuracy was significantly higher for large reward or punishment trials (*p* < 2.0 × 10^−16^) but not for different valence types (*p* = 0.53). There was no association between PCS and accuracy (*p* = 0.88). There was no effect of depression (*p*'s > 0.24) and still no effect of PCS (*p*'s > 0.20) on RT or on accuracy when HADS-D was added to the model.

### 3.4. Pain catastrophizing and MID task mood ratings

**Figure 1:**
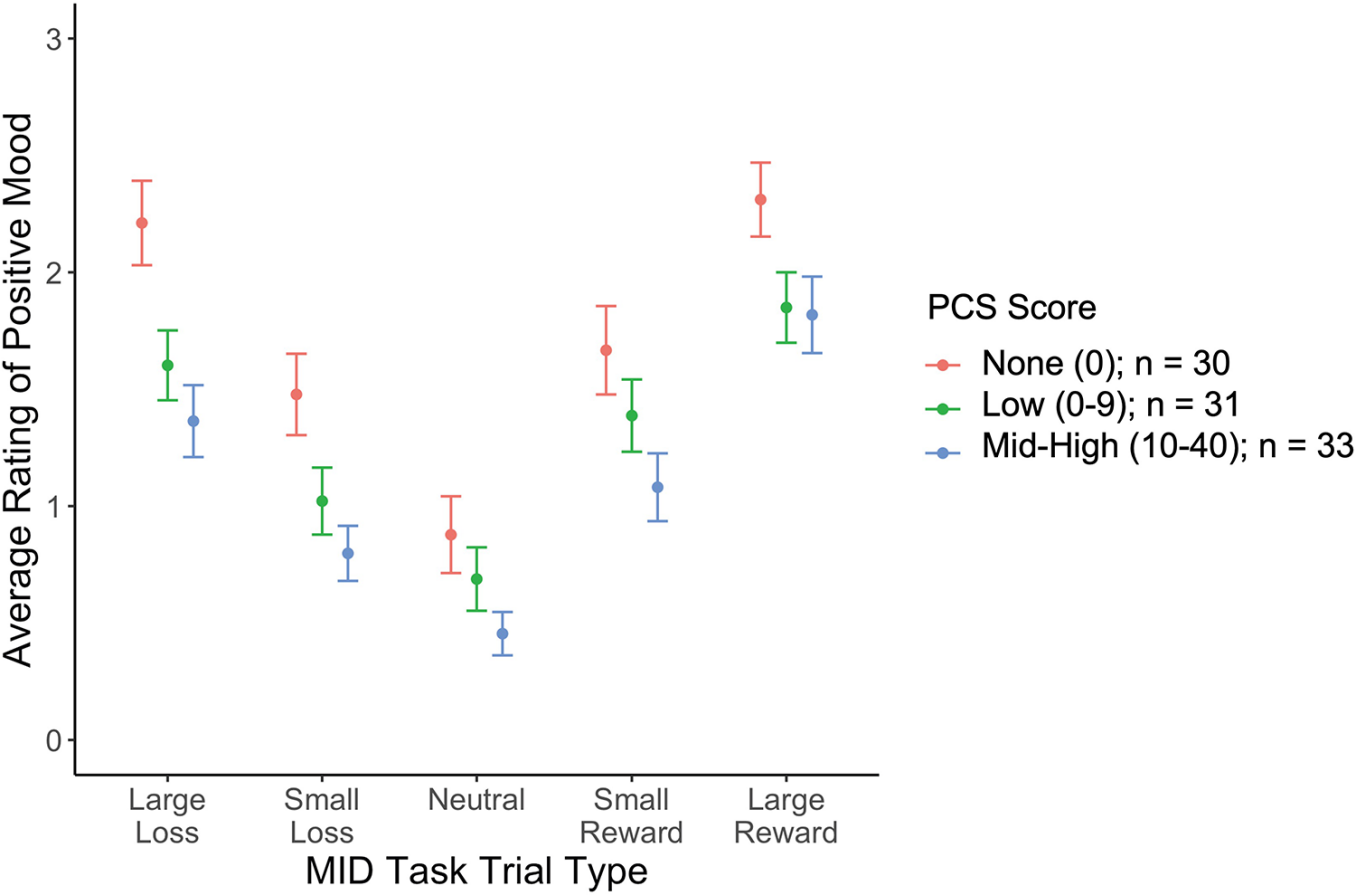
The relationship between PCS and positive mood rating by MID task trial type. PCS scores are broken into 3 categories [None (PCS = 0), Low (1–9), and Med-High (10–41)], for visualization, but were analyzed continuously in the models.

Higher PCS scores were associated with lower positive mood across all trial types (Figure 1), with no significant interaction between PCS scores and magnitude (high, low) or valence (reward, loss, neutral) of MID trial type. PCS score was not significantly associated with negative or neutral mood (Table 2). When controlling for depression, the association between PCS score and positive mood was no longer significant (stnd beta = −0.12, *t* = −1.28, *p* = 0.20).

**Table 2:** Association of PCS with post-MID task mood ratings.

	Beta	*t*-statistic	*p*-value
Positive mood			
PCS	−0.17	−2.21	0.03
Magnitude	0.31	9.6	<0.001
Valence	0.14	4.23	<0.001
Magnitude × Valence	−0.002	−0.055	0.96
Negative mood			
PCS	0.09	1.15	0.25
Magnitude	0.17	5.74	<0.001
Valence	−0.37	−12.74	<0.001
Magnitude × Valence	−0.09	−3.07	0.002
Neutral mood			
PCS	−0.08	−1.01	0.31
Magnitude	−0.08	2.15	0.03
Valence	0.11	2.77	0.006
Magnitude × Valence	0.01	0.17	0.86

Separate models were run for each mood. All predictors reported were included in each model. PCS, pain catastrophizing scale.

### 3.5. Neural responses to the MID task

**Figure 2:**
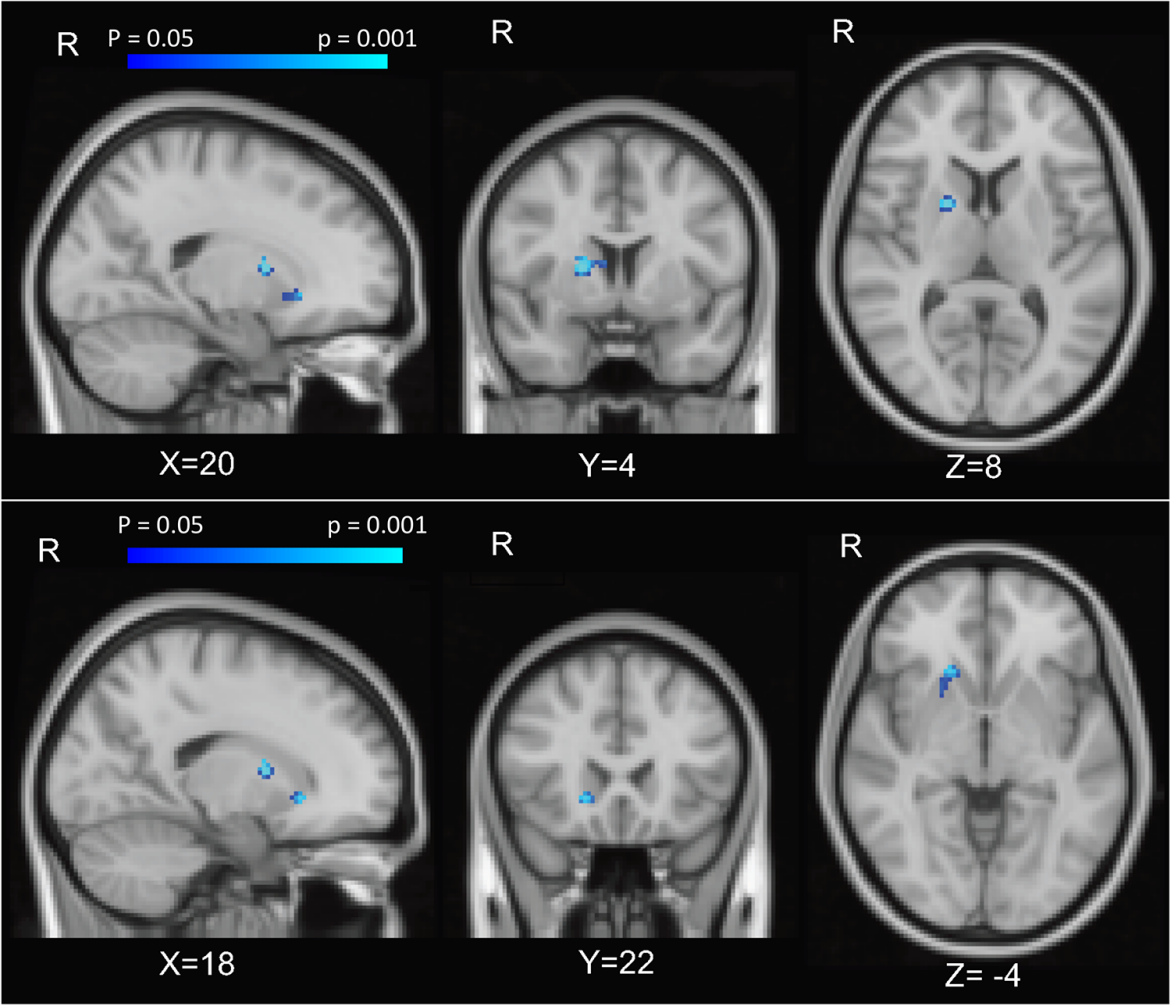
Caudate activation associated with PCS. Brain activation *z*-score maps, averaged across all participants and thresholded at FDR = 0.05, for anticipation of reward vs neutral contrast, at a threshold of *z* = 2.31.

**Figure 3:**
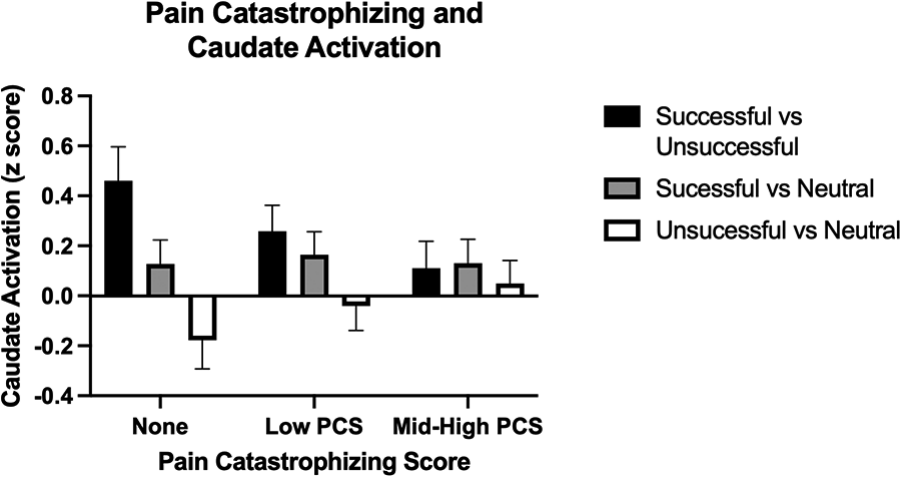
Caudate activation by trial type during the feedback phase. PCS scores are broken into 3 categories [None (PCS = 0), Low (1–9), and Med-High (10–41)], for visualization, but were analyzed continuously in the models. Error bars represent standard error of the mean.

In a voxel-wise analysis, both anticipation and feedback of reward and loss trials were associated with expected activation of the bilateral striatum, compared to no-incentive trials (Figures S1, S2). PCS was associated with two clusters within the right striatum (MNI coordinates 20, 4, 8, and 18, 22, −4) during the feedback phase (successful > unsuccessful trials) such that those with higher PCS scores had lower striatal activation (Figure 2). PCS was not significantly correlated with activation to either successful hit trials vs. neutral or unsuccessful miss trials vs. neutral, indicating that the difference between successful vs. unsuccessful trials, rather than the response to either alone, was driving this correlation (Figure 3). Neither BPI-PS nor HADS-Dep was associated with caudate or putamen activation in response to feedback for successful trials vs. unsuccessful trials. There were no clusters of brain activation associated with PCS that passed the significance threshold during the anticipation phase of the MID task.

**Figure 4:**
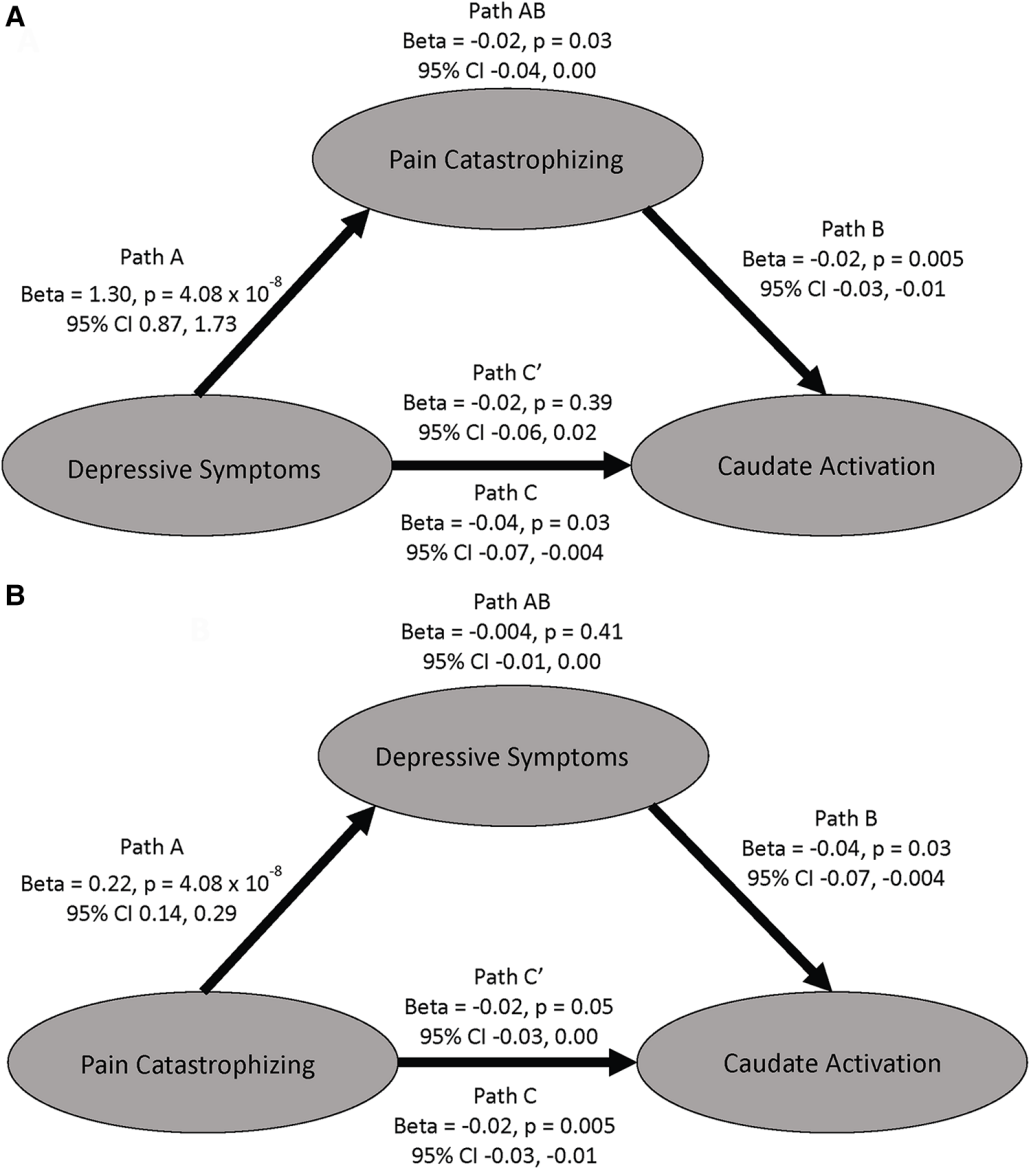
Mediation models examining the mediating effects on caudate activation to successful vs unsuccessful trials. (**A**) This model shows PCS mediating the relationship between depressive symptoms and caudate activation. (**B**) This model shows depressive symptoms do not mediate the relationship between PCS and caudate activation. All betas are unstandardized.

Due to the high behavioral correlation between PCS and HADS-Dep seen in this sample and previous findings of an association between blunted activation to the MID task and depression (48–50), we performed a mediation analysis to test the specificity of our finding to pain catastrophizing. First, in an ROI analysis with extracted COPEs from the right caudate, we found a negative association between activation to feedback of successful > unsuccessful trials and HADS-Dep scores (*r* = −0.22, CI = −0.02 to −0.41, *p* = 0.03). The relationship between HADS-Dep and caudate activation was not significant when PCS was added to the model (beta = −0.02, *p* = 0.39, CI = −0.06, 0.02, Figure 4A, path C') and PCS fully mediated the relationship between HADS-Dep and caudate activation (indirect effect = −0.02, *p* = 0.03, 95% CI: −0.04, 0.00, Figure 4A, path AB). HADS-Dep did not significantly mediate the relationship between PCS and right caudate activation (indirect effect = −0.004, *p* = 0.41, 95% CI: −0.01, 0.00). Therefore, PCS is uniquely associated with right caudate activation, independent from HADS-Dep.

We ran two sensitivity analyses. First, to further visualize the association between PCS and activation, we split the sample according to whether participants showed negative or positive activation to successful > unsuccessful trials. Those with positive scores (i.e., subjects with more activation of the right caudate on successful vs. unsuccessful trials) had lower PCS scores (*M* = 6.5) than the group with negative activation (*M* = 10.8). The groups did not differ on HADS-Dep (*p* > .05). BPI-PS did not correlate with activation in the right caudate (*p* > 0.05). Second, we ran a sensitivity analysis including the 10 participants in the sample who currently met criteria for major depressive disorder or a major depressive episode, and the 51 participants who have never had a major depressive episode. The individuals with current depression showed significantly greater PCS scores (*M* = 17.20) than those without depression (*M* = 5.80, *t* = −2.60, *p* = 0.03). There was no difference between the groups (MDD vs. no MDD) in activation to successful vs. unsuccessful trials in the right caudate (*t* = 0.61, *p* = 0.55). Thus, current depression status did not account for the association between PCS and caudate activation.

## 4. Discussion

In this sample of 94 individuals, who reported a range of conditions including pain, insomnia, and anxiety and depression symptoms, we found that pain catastrophizing, as assessed with the PCS, was associated with decreased activation in the right caudate on a task designed to elicit activation of the reward system. Mediation analyses indicated that this relationship between pain catastrophizing and caudate activation was distinct from an effect of depression on reward activation. Thus, pain catastrophizing appears to be an important variable to consider when assessing brain mechanisms of reward. Higher pain catastrophizing was also associated with decreased positive mood ratings, though not negative mood ratings, of the MID cues, consistent with prior studies suggesting that individuals high in pain catastrophizing experience and express dampened positive affective responses (51).

Individuals with pain often experience negative emotional states associated with chronic pain that increase the incidence of comorbidities like anxiety and anhedonia (52, 53), which is reflective of the well-established relationship between pain and negative affect that we also observed in this study. Clinical and preclinical data also show that relief of affective component pain is reflected by activation of reward circuits (27, 54, 55). In the current study, we found that while catastrophizing and depression were correlated, only catastrophizing was consistently associated with reduced activation in the right caudate to feedback of successful vs. unsuccessful trials. Thus, pain catastrophizing may be an important contributing factor to consider when studying affective components of pain that may affect brain circuitry.

Decreased striatal activation has been reported in studies of chronic pain patients (56–58), almost exclusively during the period of anticipation to reward or loss (56, 58). These studies implicate dysregulation in the striatum and other reward regions, such as the prefrontal cortex (PFC) (58). We attempted to replicate associations between mPFC activation on the MID task and pain catastrophizing but found no significant associations in the anticipation or outcome phase. We note that these previous studies compared groups of individuals with and without chronic pain, and did not include catastrophizing as a main outcome. In our analyses, rather than comparing those with vs. without pain, we included participants with a full range of pain scores and analyzed the PCS continuously. Thus, it is possible that dampened anticipatory activation in the PFC to the MID is a characteristic of chronic pain patients, but may not directly relate to pain catastrophizing. It is also worth noting that these previous studies generally had small sample sizes, which could lead to inflated effect sizes, reiterating the need for continued work to replicate these findings with larger sample sizes (59).

### 4.1. Limitations

Although the sample size (*N* = 94) is larger than most previous neuroimaging studies examining pain catastrophizing, replication of these results in larger samples is needed. Additionally, the study was cross-sectional so we are unable to comment on the timing of the development of depressive symptoms, PCS, and reward activation. Relatedly, we were unable to fully disentangle the specific correlations due to pain catastrophizing vs. depression, as these two constructs were highly correlated in our sample. Finally, the associations reported here need to be replicated in other samples including chronic pain samples.

Overall, catastrophizing was consistently associated with reduced reward-related caudate activation during a monetary task (i.e., those higher in catastrophizing showed less caudate activation on feedback of successful trials), and its effects were somewhat diminished when controlling for depressive symptoms, suggesting a co-influence of these inter-related variables on reward processing. This finding may represent a shared association of pain catastrophizing and depression on the reward system. This work further underscores the importance of the reward system in our understanding of the processing of pain. Future pain research should consider the inclusion of pain catastrophizing measures to continue to parse the relationship between pain, catastrophizing, and depression, to better understand if these constructs confer unique or joint effects on caudate activation in response to rewards.

## Data Availability

The raw data supporting the conclusions of this article will be made available by the authors, without undue reservation.
