# Supplementary material for: Pain catastrophizing is associated with reduced neural response to monetary reward
Source: Front Pain Res (Lausanne). 2023 Sep 7;4:1129353. doi: 10.3389/fpain.2023.1129353 (PMC10512714; doi:10.3389/fpain.2023.1129353)
Supplement: Supplementary file 1 [file Datasheet1.pdf]

# Supplemental Figures

S1 Neurosynth “Reward” Mask

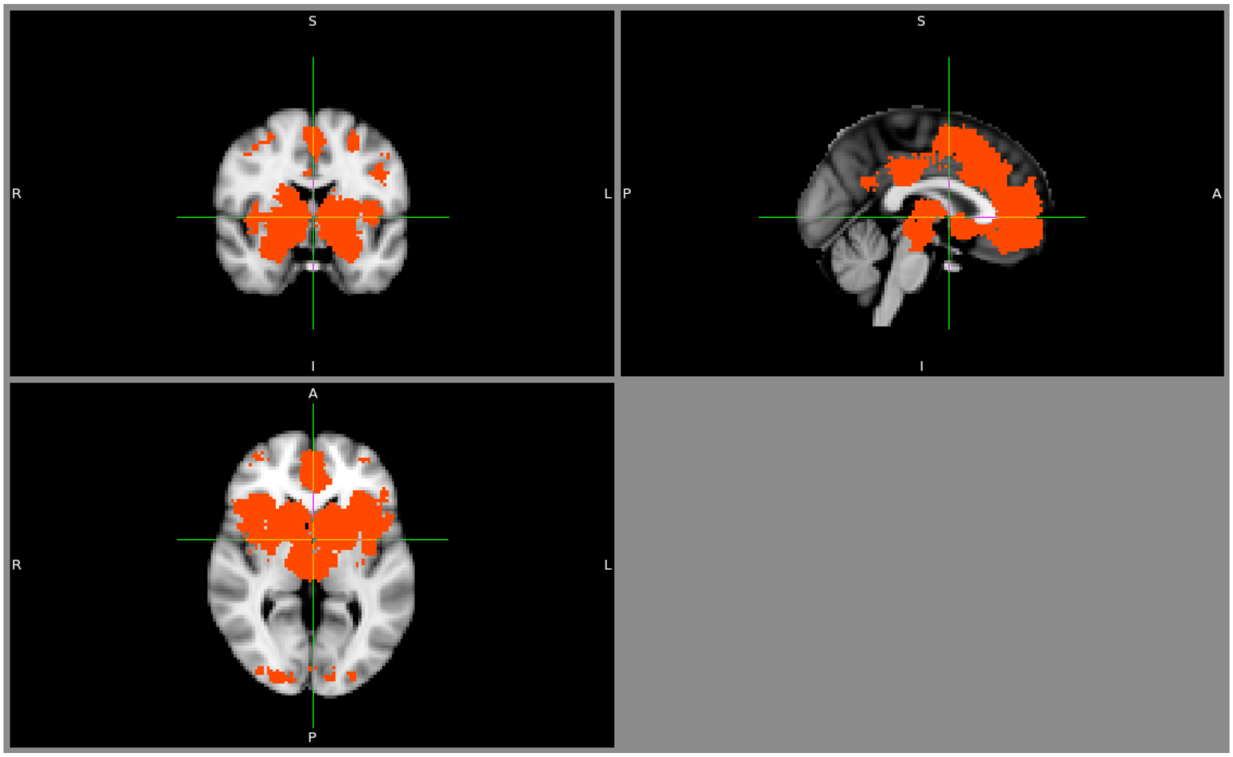


Figure S1. A reward mask obtained from NeuroSynth (reward_uniformity-test_z_FDR_0.01.nii, and used in all subsequent analyses.

S2. MID task activation to Anticipation Reward vs Neutral

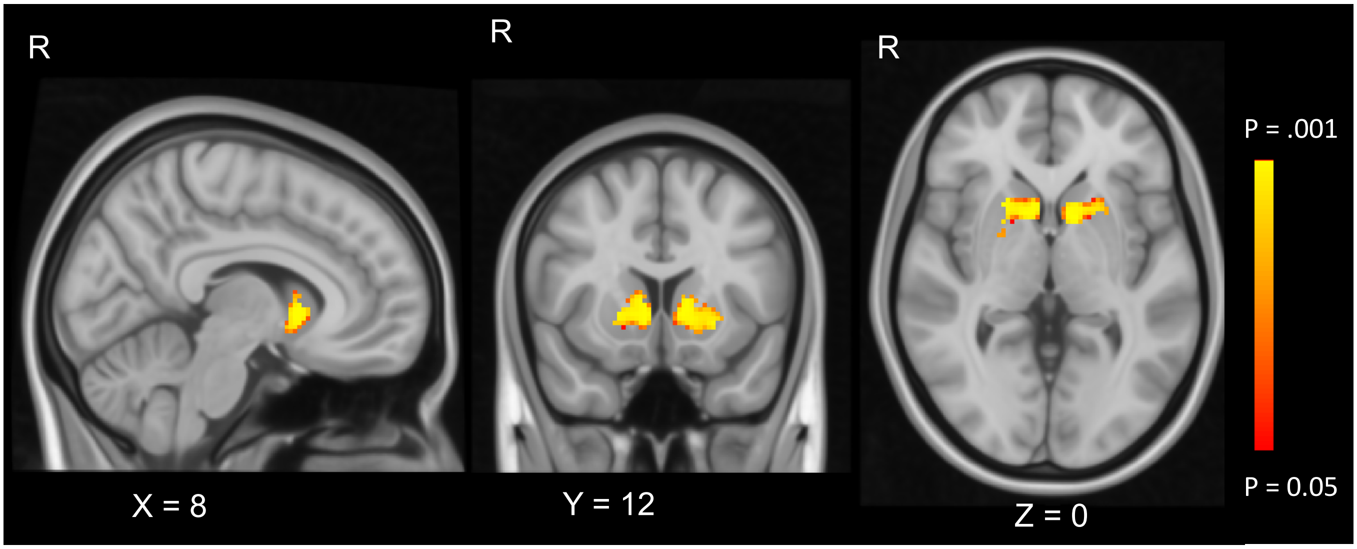


Figure S2. Brain activation z-score maps, averaged across all participants and thresholded at FDR=0.05, for the anticipation of reward vs neutral contrast if the MID task.

S3 – MID task activation to Anticipation Loss vs Neutral

S3. MID task activation to Anticipation of Loss vs Neutral

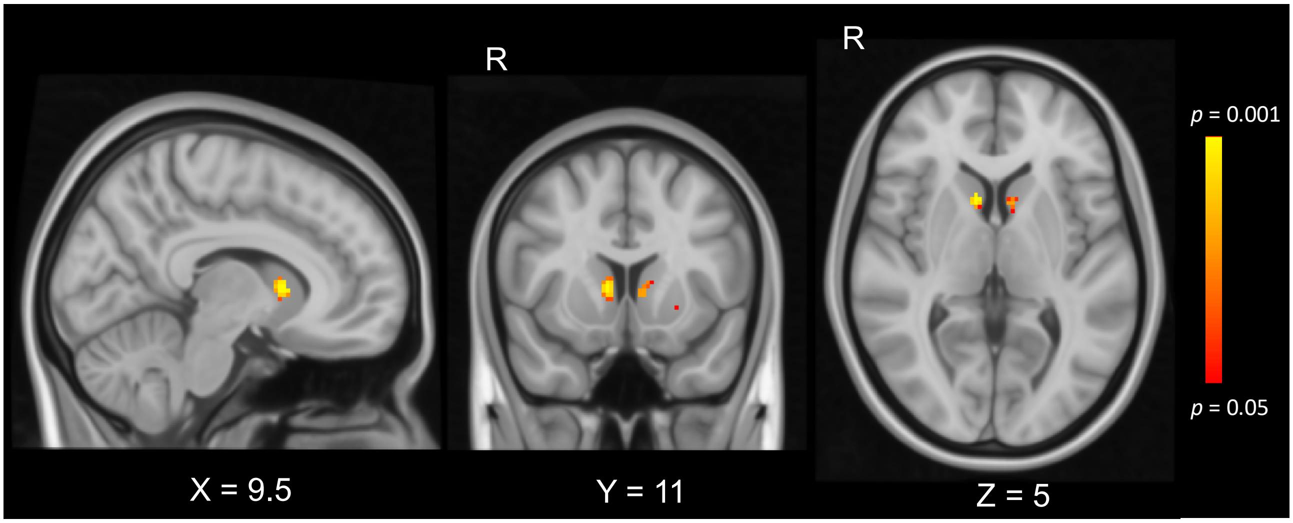


Figure S3. Brain activation z-score maps, averaged across all participants and thresholded at FDR=0.05, for anticipation of loss vs neutral contrast if the MID task.
